# Design and Analysis of a Light-Operated Microgripper Using an Opto-Electrostatic Repulsive Combined Actuator

**DOI:** 10.3390/mi12091026

**Published:** 2021-08-27

**Authors:** Jiahan Huang, Chengbin Jiang, Guanghui Li, Qinghua Lu, Haichu Chen

**Affiliations:** 1 School of Mechatronic Engineering and Automation, Foshan University, Foshan 528225, China; jiahan1989@126.com (J.H.); 2112051020@stu.fosu.edu.cn (C.J.); qhlu@fosu.edu.cn (Q.L.); 2 Shanghai Marine Equipment Research Institute, Shanghai 200031, China; liguanghui211@126.com

**Keywords:** PLZT, light-driven, microgripper, manipulation, opto-electrostatic repulsive actuator

## Abstract

The microgripper plays a critical role in micromanipulation systems; however, the handling accuracy of traditional driving microgrippers suffers from external vibration due to requiring connecting wires for an external power supply. By contrast, light driving has many advantages of remote non-contact manipulation, wireless energy transfer and no induced electromagnetic noise. In this study, an opto-electrostatic repulsive combined driving mechanism was proposed, and then a novel light-operated microgripper that used an opto-electrostatic repulsive actuator was designed and simulated. The static performance of the light-operated microgripper was investigated via simulation and numeric calculation results. The overall size of the microgripper was 1.3 mm × 0.7 mm × 1.027 mm, and the micro-objects ranging from 0 to 1000 μm in size could be manipulated and held using light. The proposed microgripper had many outstanding characteristics, such as a larger stroke, high response speed, remote non-contact manipulation, easy to integrate with an integrated circuit (IC) process and free from external interference. In addition, the dynamic control experiments of the photo-induced voltage of the PbLaZrTi (PLZT) ceramic were carried out, which shows that a stable electrical field could be obtained using the effective control methods that were developed.

## 1. Introduction

Micromanipulation in biological and biomedical engineering and the microassembly of micro-objects in microelectromechanical systems are emerging research fields with great challenges but their own promising and broad futures in this century. The accuracy, stability and dexterity of manipulation in the microscale field depend on the performance of miniaturized grippers, which are in direct contact with the manipulated micro-objects.

In micromanipulation systems, the actuation of microgrippers plays a critical role in the whole operation performance. According to the application fields, the microgrippers can be driven by various types of manipulation systems, such as piezoelectric [1,2,3,4,5,6], electromagnetic [7,8], electrothermal [9,10,11], electrostatic [12,13], ionic polymer–metal composite (IPMC) [14,15,16,17], light-driven liquid-crystal material [18] and shape memory alloy systems [19,20]. However, the microgrippers in all these cases need to be connected to a power supply via wires, which will inevitably cause electromagnetic noise and external disturbances. By contrast, the optical drive mechanism has great advantages in the micro-actuator field due to its unique characteristics of remote non-contact control, wireless energy transfer and no induced electromagnetic noise.

The lead lanthanum zirconate (PLZT) ceramic, which is based on the photostrictive effect, can transform optical energy directly into mechanical energy to achieve optical driving. However, our previous experimental results indicated that there exist a hysteresis phenomenon and slow response of the photo-induced deformation, which is due to the temperature elevation caused by the photothermal effect [21]. This has seriously hampered further PLZT ceramic developments and applications in MEMS. However, a high photovoltage of several kilovolts per centimeter is produced between the two electrodes of PLZT ceramic under the irradiation of ultraviolet light (near 365 nm). The photo-induced electric field can be used to activate the electrostriction and electrostatic drive device, based on which, the PLZT ceramic is regarded as an energy-supplying device and can indirectly achieve optical operation. The electrostatic actuators comprising parallel plate actuators [22] and vertical comb-drive actuators [12] are used in a wide variety of applications due to their easy compatibility with IC fabrication technology, high response speed, simple structure and low energy consumption. However, the electrostatic attractive actuators suffer from the “pull-in” effect that leads to structural instability and a limited stroke, which are the major problems restricting its applications. To solve these problems, some researchers reported an electrostatic repulsive-force actuator, which can eliminate the “pull-in” effect and achieve a large stroke [23], but the electrostatic structure needs a higher driving voltage. The weakness that is inherent in the electrostatic drive prevents the electrostatic actuator from large-scale commercial applications. To solve the above problems, a novel light-operated mode is presented in this paper, where the electrostatic repulsive-force actuator is actuated by the photovoltage generated by the PLZT ceramic, which can avoid the problem of requiring a high driving voltage.

The opto-electrostatic repulsive combined driving mechanism not only avoids using a higher driving voltage for electrostatic driving but also accomplishes the dominance complement of electrostatic repulsive-force driving and the high photo-induced voltage of PLZT ceramic. The light-operated microgripper based on the opto-electrostatic repulsive combined actuator has several advantages, such as remote non-contact manipulation, a fast response speed and no electromagnetic interference, and makes up for the shortcomings of traditional driving types, especially for micromanipulation in special independent environments, such as a high or a low vacuum. 

In this study, a novel light-operated microgripper driven using an opto-electrostatic repulsive actuator and a hybrid actuation mechanism was first proposed. According to the photovoltaic/electrostatic hybrid driving mechanism, the photovoltage of the PLZT ceramic was derived in consideration of the influence of external load, by which the expression of opto-electrostatic repulsive force was gained. After that, the structural parameters of the electrostatic repulsive actuator were determined via an investigation of the relationship between the repulsive force and the structural parameters using the software COMSOL (COMSOL Multiphysics 5.6, COMSOL, Inc., Burlington, MA, USA). Then, the structure of light-operated microgripper was introduced, and the mathematical modeling of microgripper was established, by which the influences of light intensity and illumination time on the gripper displacement were analyzed. The finite element analysis was conducted to obtain the static and the dynamic performance of the opto-electrostatic repulsive microgripper. Finally, the dynamic control experiment of photovoltage was performed to offer an accurate and stable driving voltage and eliminate the residual photovoltage. 

## 2. Opto-Electrostatic Repulsive Combined Driving Model

A PLZT ceramic was used as an actuator to directly drive a microgripper based on the photostrictive effect in previous research [24]. To overcome the problems of a slow response speed and serious hysteresis phenomenon, a novel opto-electrostaic combined driving mechanism was proposed. The several kilovolts per centimeter of electrical field produced between the electrodes of PLZT ceramic that were irradiated by ultraviolet light was applied to the electrostatic actuator, causing it to move. 

### 2.1. The Model of Photo-Induced Electrical Field with Loads

**Figure 1:**
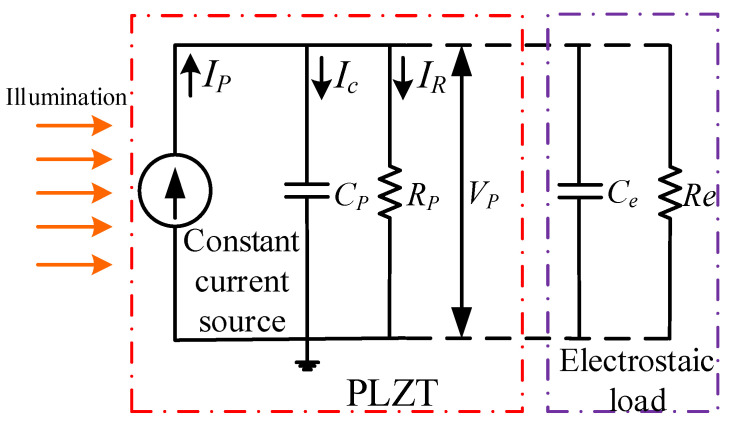
Equivalent electrical model for photovoltaic/electrostatic hybrid driving.

The photo-induced voltage of the PLZT ceramic was discussed under consideration of the influence of the elevated temperature caused by impure UV light in our previous study [19]; however, the load effect and the coupling problem have not been explored. To simplify the model, the photothermal effect, thermal expansion effect and pyroelectric effect caused by impure UV light were ignored; thus, the photo-induced voltage was thought of as a consequence of an anomalous photovoltaic effect. The PLZT ceramic under the illumination of UV light could be viewed as a parallel circuit of the current source; therefore, the equivalent electrical model of the PLZT ceramic under load is shown in Figure 1. 

According to the equivalent electric model shown in Figure 1, the PLZT ceramic could be viewed as a parallel circuit of the current source, resistance *R_p_* and the capacitance *C_p_*, and similarly, the electrostatic load could be also viewed as a parallel circuit of resistance *R_e_* and capacitance *C_e_*. Thus, the photovoltage generated by the PLZT ceramic could be expressed as follows:

(1) $$V_{p}=I_{p}R(1-e^{-\frac{t}{RC}})$$

where $R=\frac{R_{p}R_{e}}{R_{p}+R_{e}}$, $C=C_{p}+C_{e}$, *R_e_* and *C_e_* are the equivalent resistance and capacitance of the electrostatic load; *I_p_* is the photocurrent.

Because the air conductivity is generally 5 × 10^−14^ S/m, the resistance of the electrostatic actuator is on the order of 10^16^ Ω, which is much larger than the photo-resistance of the PLZT ceramic. Furthermore, because the relative permittivity of air is about 1, which is much less than the permittivity of the PLZT ceramic (about 1860), the air capacitance of the electrostatic actuator can be negligible. Thus, the effect of the equivalent resistance and capacitance of electrostatic actuator on the photo-induced voltage were negligible; therefore, the expression of the photovoltage could be written as follows:

(2) $$V_{p}=I_{p}R_{p}(1-e^{-\frac{t}{R_{p}C_{p}}})=V_{s}(1-e^{-\frac{t}{\tau_{1}}})$$

where *V_s_* is the saturated photo-voltage and $V_{s}=I_{p}R_{p}$; $\tau_{1}$ is the illumination time constant and $\tau_{1}=R_{p}C_{p}$.

### 2.2. The Model of Electrostatic Repulsive Actuator

**Figure 2:**
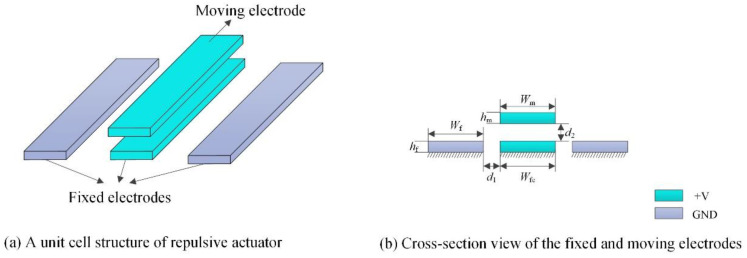
Schematic diagram of the electrostatic repulsive actuator. (**a**) A unit cell structure of repulsive actuator; (**b**) Cross-section view of the fixed and moving electrodes.

Aiming at the limitation of the electrostatic attraction structure, an electrostatic repulsive actuator is widely studied for its large stroke, higher stability and reliable structure. An elementary cell of the electrostatic repulsive actuator was composed of three fixed electrodes and a moving electrode. The three fixed electrodes were aligned side by side, and the moving electrode aligned with the central fixed electrode in the vertical direction and was supported by an anchoring spring, which is not shown in Figure 2. A voltage was applied to the moving electrodes and the central fixed electrode while grounding the other two fixed electrodes, as shown in Figure 2. The electrostatic field distribution of the moving electrode was symmetrical under the action of an applied voltage, and the strength of the electric field around the upper surface was larger than that around the lower surface. In the symmetrical field, the electrostatic forces on the two lateral surfaces of the moving electrode were equal and opposite, and the force on the upper surface is larger than that on the lower surface; therefore, the net force on the moving electrode produced a vertical upward electrostatic repulsive force, which was used to drive the microgripper. The length of each electrode was much larger than its width, height and distance between adjacent electrodes. 

According to the principle of virtual work, the electrostatic force on the moving electrode in a unit cell of an electrostatic repulsive actuator could be expressed as follows:

(3) $$F=\frac{dE}{dd_{2}}$$

where *d*_2_, as shown in Figure 2b, is the distance between the fixed and moving electrode. *E* is the energy stored in the electrostatic structure, which could be calculated using

(4) $$E=\frac{1}{2}V^{2}C$$

where *V* is the voltage applied in electrodes and *C* is the capacitance between the fixed electrodes and the moving electrodes.

By substituting Equation (4) into Equation (3), the electrostatic repulsive force could be written as follows:

(5) $$F=\frac{1}{2}V^{2}\frac{dC}{dd_{2}}$$

**Figure 3:**
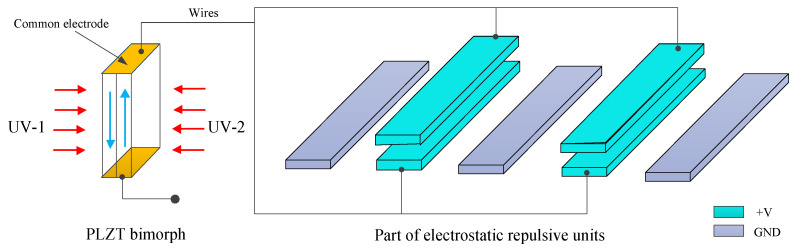
Working principle of a photovoltaic–electrostatic repulsive actuator.

To avoid having too high of a driving voltage and achieve light operation, the photo-induced voltage of the PLZT ceramic was applied to the electrostatic actuator. The schematic diagram of the working principle is shown in Figure 3. The PLZT bimorph consisted of two PLZT ceramics, which were adhered with opposing polarized directions. The common electrodes were plated on both ends of the PLZT bimorph, and the positive electrode was connected with the moving electrode and the centered fixed electrode, while the other common electrode kept the circuit states open.

When the PLZT bimorph was illuminated using UV-1, a large photovoltage in the order of several kilovolts per centimeter was excited between the two common electrodes and was applied to the electrostatic electrodes, thus the moving electrode was pushed to move away from the fixed electrodes. After that, the UV-1 was switched off and the UV-2 was turned on, which caused a reverse voltage to be generated, and therefore the residual voltage could be eliminated quickly. Thus, the prompt response and stable operation of opto-electrostatic repulsive actuator could be achieved. 

The photovoltage expressed in Equation (2) was the driving voltage of the electrostatic structure, where the influence of the electrostatic structure’s equivalent resistance and capacitance were negligible. Therefore, the expression of the combined driving force could be obtained by substituting Equation (2) into Equation (5):

(6) $$F=\frac{1}{2}{\left[V_{s}(1-e^{-\frac{t}{\tau_{1}}})+B_{1}(1-e^{-\frac{t}{\tau_{\theta }}})\right]}^{2}\frac{dC}{dd_{2}}$$

Generally, according to our previous works, the saturated voltage *V_s_*, illumination time constant $\tau_{1}$, thermal time constant $\tau_{\theta }$ and parameter $B_{1}$ can be identified through numerical fitting and simulation methods. Furthermore, the capacitance’s rate of change with respect to *d*_2_ dCdd2 can be calculated by using the polygon conformal transformation method [23]. To avoid the complexity of solving the model and the calculation error caused by this method, the effects of the electrostatic structure parameters were studied using the numerical method based on the software COMSOL Multiphysics, which is discussed in the next section.

## 3. Design and Working Principle of the Light-Operated Microgripper

### 3.1. The Structure Simulation of Electrostatic Repulsive Elementary Cell

Once the photo-induced voltage was confirmed, the structural parameters of the electrostatic repulsive part played an important role in the output displacement and grasping force of the microgripper jaw. To offer a reference for rationally determining the structural parameters in the initial stage of the electrostatic repulsive structural design, the FEA software COMSOL Multiphysics was adopted to study the influences of these parameters on the electrostatic force. As shown in Figure 2b, *d*_1_ is the lateral distance between two adjacent fixed electrodes, *d*_2_ is the vertical distance between the moving electrode and centered fixed electrode, *W*_fc_ is the width of the central fixed electrode, *W*_f_ is the width of the other two fixed electrodes and *W*_m_ and *h*_m_ are the width and height of moving electrode, respectively. Because the height of fixed electrodes was mainly determined by PolyMUMPs and had a minimal effect on the electrostatic field distribution, mainly the influences of *V*, *d*_1_, *d*_2_, *W*_m_, *W*_f_ and *h*_m_ on the electrostatic repulsive force are discussed in this section.

#### 3.1.1. Electrostatic Repulsive Force versus the Applied Voltage *V*

**Figure 4:**
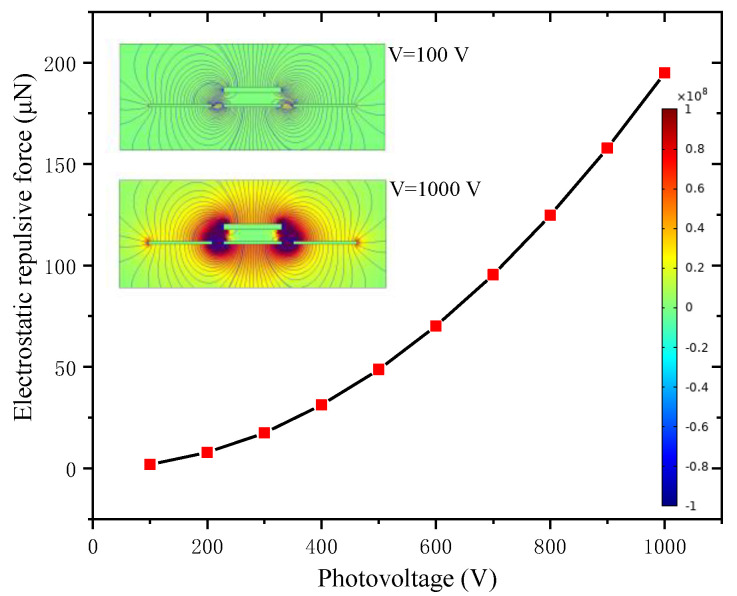
Electrostatic repulsive force versus the applied photovoltage (*d*_1_ = *d*_2_ = 4 μm, *W*_m_ = 20 μm, *W*_f_ = 22 μm and *h*_m_ = 2 μm).

**Figure 5:**
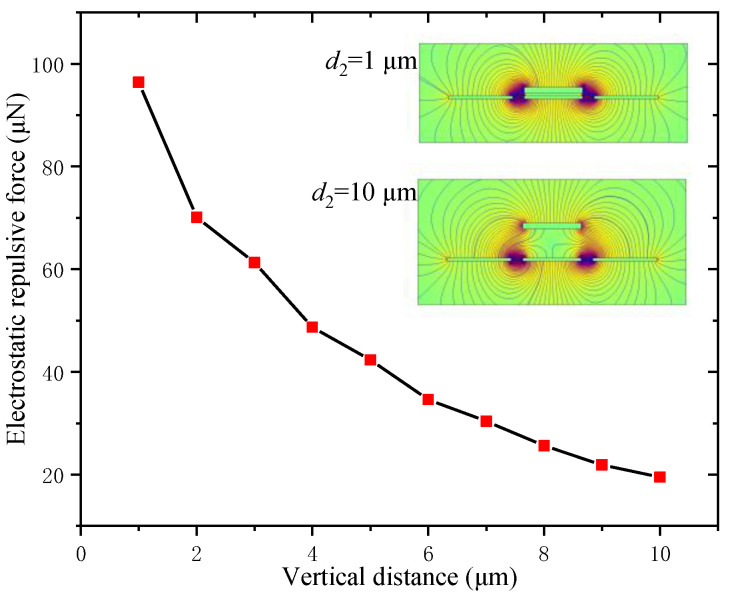
Electrostatic repulsive force versus the vertical distance between the moving electrode and the central fixed electrode (*V* = 500 V, *d*_1_ = 4 μm, *W*_m_ = 20 μm, *W*_f_ = 22 μm and *h*_m_ = 2 μm).

**Figure 6:**
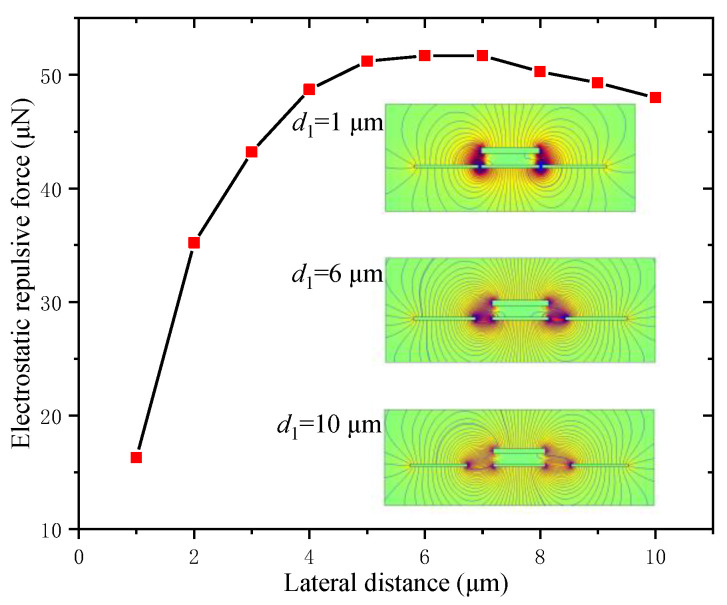
Electrostatic repulsive force versus the lateral distance between two adjacent fixed electrodes (*V* = 500 V, *d*_2_ = 4 μm, *W*_m_ = 20 μm, *W*_f_ = 22 μm and *h*_m_ = 2 μm).

**Figure 7:**
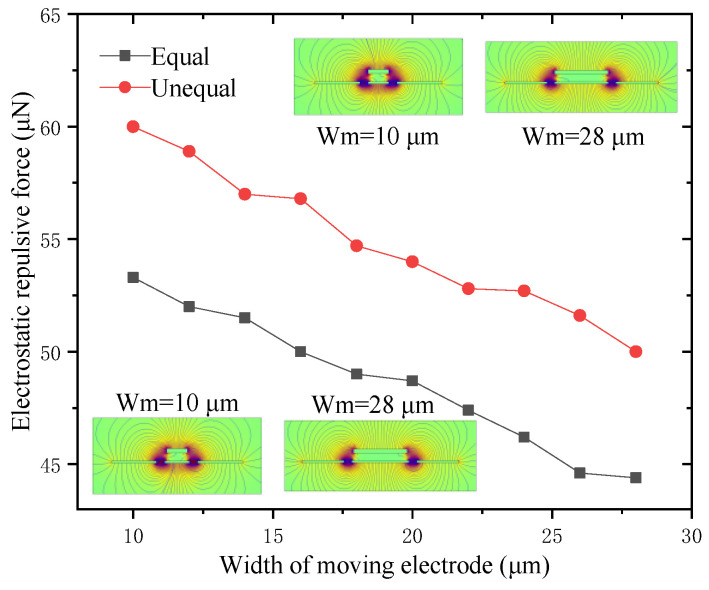
Electrostatic repulsive force versus the width of moving electrode (*V* = 500 V, *d*_1_ = *d*_2_ = 4 μm, *W*_f_ = 22 μm and *h*_m_ = 2 μm).

**Figure 8:**
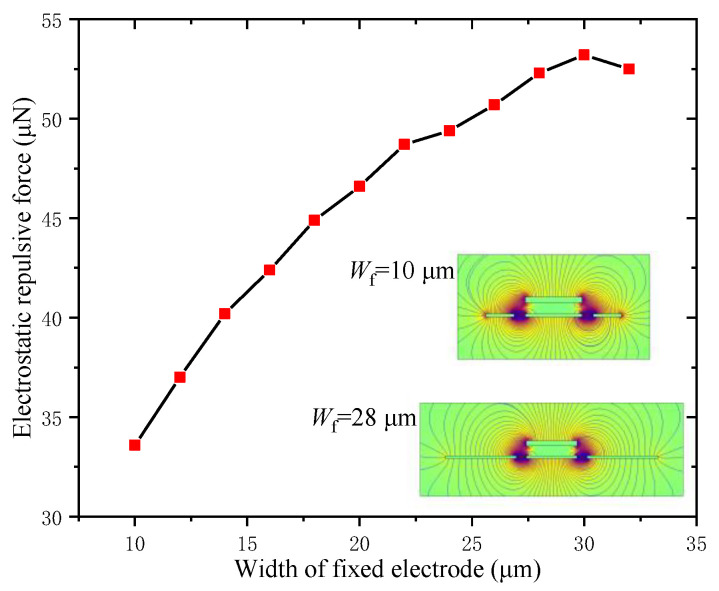
Electrostatic repulsive force versus the width of fixed electrode (*V* = 500 V, *d*_1_ = *d*_2_ = 4 μm, *W*_m_ = 20 μm and *h*_m_ = 2 μm).

**Figure 9:**
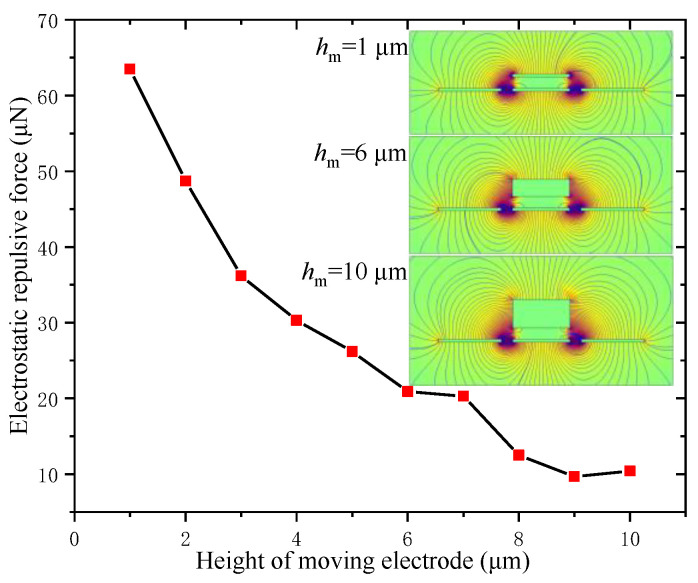
Electrostatic repulsive force versus the height of moving electrode (*V* = 500 V, *d*_1_ = *d*_2_ = 4 μm, *W*_m_ = 20 μm and *W*_f_ = 22 μm).

As discussed above, the photo-induced voltage of PLZT ceramic did not change with the structure of the electrostatic repulsive actuator; thus, the applied photovoltage was a critical factor for improving the device’s output performance. The electrostatic repulsive force versus the applied photovoltage, for *d*_1_ = *d*_2_ = 4 μm, *L* = 800 μm, *W*_m_ = 20 μm, *W*_f_ = 22 μm and *h*_m_ = 2 μm, was calculated using COMSOL Multiphysics and is plotted in Figure 4. The electrostatic field distribution under two different photovoltages is shown as the inset, and the color legend value with units of volts per meter is on the right, which is the same in Figure 5, Figure 6, Figure 7, Figure 8 and Figure 9. 

When the geometric construction was determined, the applied photovoltage was varied in the range of 100–1000 V; Figure 4 shows that the electrostatic repulsive force increased with the applied voltage and the changing trends tended to be linearly approximated with respect to the increase of voltage. However, the electrostatic attractive force grew exponentially with the increasing applied voltage in the case of a low applied voltage, and sharply increased to a maximum when the applied voltage increased beyond a certain value. Thus, the driving force could not be linearly controlled by changing the applied voltage. By contrast, the corresponding driving force could be achieved by controlling the applied voltage when the electrostatic repulsive structure was determined.

#### 3.1.2. Electrostatic Repulsive Force versus the Vertical Distance *d*_2_

Regarding the structural design of the electrostatic actuator, the electrostatic repulsive force was strongly dependent on the vertical distance between the moving electrode and the central fixed electrode. Thus, the electrostatic repulsive force versus the vertical distance for *V* = 500 V, *d*_1_ = 4 μm, *L* = 800 μm, *W*_m_ = 20 μm, *W*_f_ = 22 μm and *h*_m_ = 2 μm was calculated and is plotted in Figure 5, and the electrostatic field distributions of two different distances are shown in the upper-right corner of the graph. 

Figure 5 shows that the electrostatic repulsive force decreased with the increasing vertical distance but the trend variations decreased. The reason was that the electrostatic energy mainly concentrated in the capacitance between the moving electrode and the fixed electrode in the case of the smaller vertical distance, but the electrostatic energy was gradually transferred to the capacitance between the adjacent fixed electrodes, which did not vary with the vertical distance. The transformation of the electrostatic field distribution between the electrodes can be found in the color image in Figure 5. According to the influence rule of the vertical distance shown in Figure 5, the vertical distance should be set as low as possible under the acceptable fabrication process in the design of an electrostatic repulsive structure. 

#### 3.1.3. Electrostatic Repulsive Force versus the Lateral Distance *d*_1_

The lateral distance between two adjacent fixed electrodes was an important factor for the electrostatic repulsive force, which was calculated and is shown in Figure 6 when *V* = 500 V, *d*_2_ = 4 μm, *W*_m_ = 20 μm, *W*_f_ = 22 μm and *h*_m_ = 2 μm, and the inserted graphs give the electrostatic field distribution of three different lateral distances.

From Figure 6, the electrostatic repulsive force increased quickly when the lateral distance *d*_1_ increased from 1 to 5 μm, and then almost remained constant. This was because the electrostatic repulsive force was a comprehensive result of the electrostatic forces acting on the upper and lower surface of the moving electrode and the decreased velocity of the electrostatic force acting on the upper surface was faster than that on the lower surface when the lateral distance was smaller than 5 μm. In contrast, when the lateral distance was greater than 7 μm, the electrostatic repulsive force decreased slowly, which was caused by the changes in the distribution of the electric field. Therefore, in order to obtain the largest electrostatic repulsive force, the lateral distance was determined to be 6 μm.

#### 3.1.4. Electrostatic Repulsive Force versus the Width of the Moving Electrode *W*_m_

The width of the moving electrode *W*_m_ also had a great influence on the performance of the electrostatic repulsive actuator. Two kinds of cases for *V* = 500 V, *d*_1_ = *d*_2_ = 4 μm, *W*_f_ = 22 μm and *h*_m_ = 2 μm were calculated, where the width of the moving electrode was equal to or unequal to that of the central fixed electrode. The calculation results are shown in Figure 7, and the inserted graphs show the electrostatic field distributions of different widths of the moving electrode.

It is obvious from Figure 7 that the electrostatic repulsive force decreased linearly with the increase of the width of moving electrode *W*_m_, and the electrostatic repulsive force with the unequal width was larger than that with the equal width. The reason for this was that the larger width of the central fixed electrode could provide a stronger electrostatic field. According to the calculation results, the smaller width of the moving electrode caused a larger electrostatic repulsive force; however, a too narrow moving electrode led to structural instability, which could easily cause the moving electrode to tilt and collapse. As a consequence, both the greater electrostatic repulsive force and the stability of the structure should be considered in our design, and the widths of the moving electrode and the central fixed electrode were chosen as 15 μm and 17 μm, respectively.

#### 3.1.5. Electrostatic Repulsive Force versus the Width of the Fixed Electrode *W*_f_

The width of the fixed electrode *W*_f_ had an influence on both the electrostatic repulsive force and the overall structural dimension of the actuator. The influence rule of *W*_f_ was calculated and analyzed for *V* = 500 V, *d*_1_ = *d*_2_ = 4 μm, *W*_m_ = 20 μm and *h*_m_ = 2 μm, and the graphs of electrostatic field distribution of different widths of fixed electrodes are shown in Figure 8.

Figure 8 shows that the electrostatic repulsive force increased approximately linearly during the course of the width of the fixed electrode *W*_f_ increasing from 10 μm to 30 μm, and then decreased. Thus, there was a maximum value of the electrostatic repulsive force when the width of the fixed electrode *W*_f_ was 30 μm. Therefore, the widths of the other two fixed electrodes were determined as 30 μm in the structural design. 

#### 3.1.6. Electrostatic Repulsive Force versus the Height of the Moving Electrode *h*_m_

The height of the moving electrode *h*_m_ not only affected the electrostatic repulsive force but also the strength of the actuator. The electrostatic repulsive force versus the height of moving electrode was calculated for *V* = 500 V, *d*_1_ = *d*_2_ = 4 μm, *W*_m_ = 20 μm and *W*_f_ = 22 μm, and the results are shown in Figure 9. The inserted graphs show the electrostatic field distribution with different heights of the moving electrode, namely, *h*_m_ = 1 μm, 6 μm and 10 μm. 

The electrostatic repulsive force decreased quickly with the increase in the height of the moving electrode; therefore, a lower driving voltage was needed and a greater displacement could be obtained if a smaller height of the moving electrode was used. However, this resulted in a decrease in the yield strength and, hence, it collapsed more easily. Thus, the structural design must synthesize the consideration for the structural stability, reliability, greater output displacement and the lower voltage. 

The initial vertical distance between the moving electrode and the central fixed electrode *d*_2_ and the thickness of the fixed electrodes *h*_f_ were mainly determined by the microfabrication process. Thus, according to the PolyMUMPS standard commercial fabrication process, *d*_2_ = 1 μm and *h*_f_ = 0.5 μm were chosen. From the above calculation results, by taking the standard commercial fabrication process into consideration, the structural parameters of the electrostatic repulsive actuator shown in Figure 2b were determined and are listed in Table 1. 

### 3.2. The Structure Design of Microgripper

#### 3.2.1. Principle of Operation

**Figure 10:**
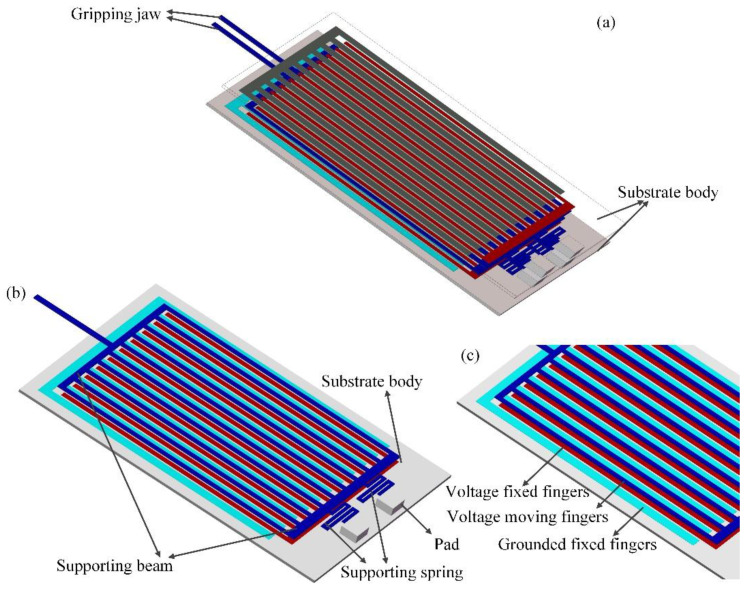
Schematic detailed view of the microgripper. (**a**) Structural diagram of the whole device; (**b**) top view of one microgripper arm; (**c**) closer view of the electrostatic repulsive actuator configuration.

Figure 10 illustrates the mechanism of the light-operated microgripper system, which consisted of two symmetrical gripper arms, a supporting beam, a substrate body, a supporting spring, PLZT ceramics (not shown in Figure 10) and anchoring pads. The gripper arms were supported by many moving electrodes and a supporting beam, which were anchored to the substrate body through two serpentine springs. Each unit of the electrostatic repulsion actuator consisted of a moving electrode and three fixed electrodes on the substrate body, where the moving electrode was aligned with the central fixed electrode, as shown in Figure 2. When a voltage generated by the PLZT ceramic under the irradiation of ultraviolet light was applied to the moving electrode and the central fixed electrode while the other two fixed electrodes were grounded, an electrostatic repulsive force acting on the moving electrode was generated and pushed the moving electrodes upward. Therefore, the generated opto-electrostaic repulsive force resulted in a bending motion on the movable electrodes, which was eventually amplified to the microgripper arm tip to manipulate the target objects. The two microgripper arms and the respective actuators were symmetric, and the initial gap between the two arm tips was 1000 μm. The overall dimensions of the proposed light operated microgripper were 1300 μm × 700 μm × 1027 μm. The micro-objects with sizes ranging from 0 to 1000 μm could be remotely manipulated without contact using the proposed light-operated microgripper. 

The 3D view of the microgripper and the closer detailed view of the electrode configuration are shown in Figure 10a,c, respectively. The two PLZT ceramics were bonded separately to the outside of the substrate body. The wire connection mode between the PLZT ceramics and the electrodes of electrostatic repulsive unit was conducted as shown in Figure 3. The two PLZT ceramics were adhered in opposing polarized directions. When one PLZT ceramic was illuminated by UV-1 light, a high photovoltage was generated between the common electrodes of the PLZT ceramics, which was applied on the electrostatic repulsive units; thus, the gripper arms were actuated to grasp the micro-object. By activating the microgripper using UV light, we were able to optically and remotely operate the micro-objects without contact. After gripping and transferring the object to the desired location, the UV-1 was turned off and the other PLZT ceramic was irradiated by UV-2 light, a reverse photovoltage is produced to eliminate the forward residual voltage, and then the micro-object was released due to the flexure beam’s reaction force. The main parameters of the electrostatic repulsive unit were determined via simulation and optimal design, which are shown in Table 1.

#### 3.2.2. The Output Displacement Model of the Microgripper

According to the mechanism of the opto-electrostatic repulsive actuator and the geometric construction of microgripper proposed in the previous section, a model of the light-operated microgripper is presented, which was used to predict its manipulation performance. The model was established based on the photovoltage output of the PLZT ceramic with a load under the irradiation of UV light and the relationship between the applied voltage and the arm tip displacement.

**Figure 11:**
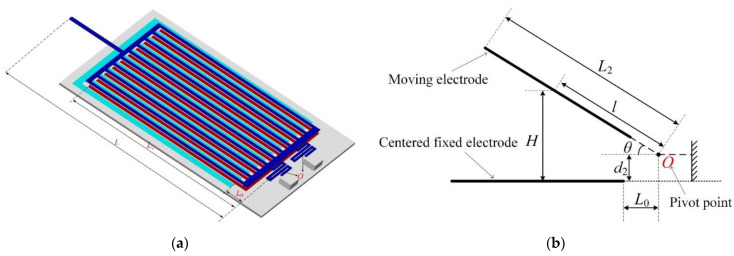
Principle view of the torque generated in the electrostatic repulsive actuator. (**a**) Dimension diagram of microgripper; (**b**) Side view of the electrostatic repulsive actuator.

As mentioned above, the bending of the gripper arms was obtained due to the torque generated in each electrostatic repulsive driving unit. The geometry and parameters of the novel opto-electrostatic repulsive microgripper are shown in Figure 11a. Figure 11b illustrates the schematic diagrams of the torque generated in the electrostatic repulsive driving unit, which caused the rotation of moving electrodes around the center of the supporting spring. As shown in Figure 11, the torque generated by the electrostatic repulsive force in a cross-section could be obtained as follows:

(7) $$T=\int_{L_{0}}^{L_{2}}F_{cross}(H)\cdot l\cdot \cos(\theta )\cdot dl$$

where *L*_0_ is the horizontal distance from the pivot point *O* to the central fixed electrode and *L*_2_ is the distance from the pivot point *O* to the ends of the moving electrodes. *θ* is the angle between the moving electrode and the fixed electrode, which was determined using the bending deformation of the moving electrode under the electrostatic repulsive loading. $F_{cross}(H)$ is the repulsive force in a cross-section of the electrodes. *H* is the vertical distance between the moving electrode and the central fixed electrode and could be expressed as $H=l\sin(\theta )+d_{2}$.

According to a previous study [23], the electrostatic repulsive force acting on the moving electrode $F_{cross}(H)$ could be expressed as follows:

(8) $$F_{cross}(H)=V^{2}NL\frac{dC_{cell}}{dH}=V^{2}NLf_{unit}(H)$$

where $f_{unit}(H)$ is the force produced in a cross-section of the unit cell per moving electrode with a driving voltage of 1 V. *V* is the applied driving voltage provided by the PLZT ceramic, *N* is the number of the moving electrodes and *L* is the length of the moving electrodes.

**Figure 12:**
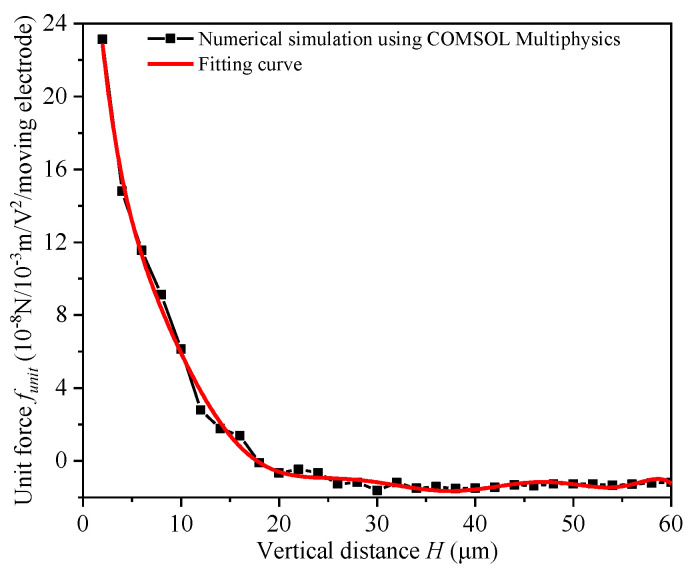
The unit force per moving electrode in a cross-section versus the vertical distance.

The electrostatic repulsive force can be calculated using polygon conformal transformation method, but it is very complicated. Therefore, the repulsive force of an electrostatic repulsive unit with the physical dimension shown in Table 1 was simulated using COMSOL Multiphysics software, where the relationship of the simulated force (*L* = 1000 μm, *V* = 1 V and *N* = 1) and the different vertical distances could be obtained and are shown in Figure 12. Figure 12 shows that the electrostatic repulsive became electrostatic attraction force when the vertical distance between the moving and the central fixed electrode reached a certain value; therefore, the driving displacement of the electrostatic repulsive actuator was larger than that of electrostatic attraction, but also had limitations. Then, the simulation curves could be approximated to a polynomial expression by using the least-squares method; thus, the function $f_{cross}(h)$ could be written as follows:

(9) $$f_{cross}(h)=A_{0}+A_{1}H+A_{2}H^{2}+A_{3}H^{3}+A_{4}H^{4}+A_{5}H^{5}+A_{6}H^{6}+A_{7}H^{7}+A_{8}H^{8}+A_{9}H^{9}$$

where the coefficients in Equation (9) were found to be *A*_0_ = 3.713 × 10^−7^, *A*_1_ = −9.804 × 10^−8^, *A*_2_ = 1.662 × 10^−8^, *A*_3_ = −1.81 × 10^−9^, *A*_4_ = 1.196 × 10^−10^, *A*_5_ = −0.483 × 10^−11^, *A*_6_ = 1.198 × 10^−13^, *A*_7_ = −1.783 × 10^−15^, *A*_8_ = 1.46 × 10^−17^ and *A*_9_ = −5.047 × 10^−20^.

It should be noted that these coefficient values were only applicable for the structural parameters proposed in this paper. The simulation and the numerical fitting should be rerun if the structural parameters change.

Substituting Equations (9) and (8) into Equation (7), the torque acting on the gripper arms could be obtained as follows:

(10) $$T=10^{-3}V^{2}NL\cos\theta \sum_{i=0}^{i=9}A_{i}\int_{L_{0}}^{L_{2}}l{\left(l\sin\theta +d_{2}\right)}^{i}dl$$

Because of the stiffness calculation complexity of the supporting beam, ANSYS software (COMSOL Multiphysics 5.6, COMSOL, Inc., Burlington, MA, USA) was used to estimate the stiffness. In the finite element analytical model, whose structural parameters are shown in Table 1, equal forces were applied to the ends of the moving electrodes and the moving electrodes were bent as a result. The electrostatic repulsive force acting on the moving electrodes was a uniformly distributed load (ignoring the effect of a fringe field), where the distributed loading was simplified as a concentrated force at the tip of the moving electrode, which will result in errors, but the stiffness approximation will be corrected using the experimental results in a later study.

**Figure 13:**
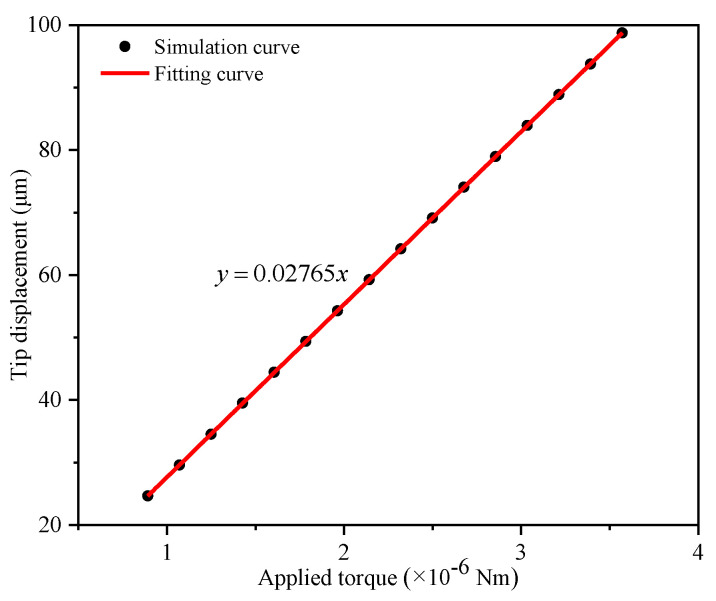
Tip displacement of the microgripper versus the applied torque on the moving electrode.

According to the simulation results, the relationship with different applied torques between the tip displacement of the gripper arm and the applied torque was obtained, and then the simulation curve was fitted with a linear function using the least-squares method, which is shown in Figure 13. Thus, the relationship between the applied torque and the tip displacement of the microgripper was as follows:

(11) $$C_{T-D}=0.1275m/(\times 10^{-7}N\cdot m)$$

where *C_T − D_* is the converting coefficient.

Therefore, the gripping displacement of the microgripper could be expressed as follows:

(12) $$D=T\cdot C_{T-D}$$

where *D* is the tip displacement of the gripper arm and *T* is the total torque generated by the electrostatic repulsive force.

Substituting Equation (10) into Equation (12), the output displacement of the microgripper could be acquired as follows:

(13) $$D=10^{-3}V^{2}NLC_{T-D}\cos\theta \sum_{i=0}^{i=9}A_{i}\int_{L_{0}}^{L_{2}}l{\left(l\sin\theta +d_{2}\right)}^{i}dl$$

Rearranging Equation (13), the expression of the applied driving voltage could be written as:

(14) $$V=\sqrt{\frac{10^{3}D}{NC_{T-D}L\cos\theta \sum_{i=0}^{i=9}A_{i}\int_{L_{0}}^{L_{2}}l{\left(l\sin\theta +d_{2}\right)}^{i}dl}}$$

**Figure 14:**
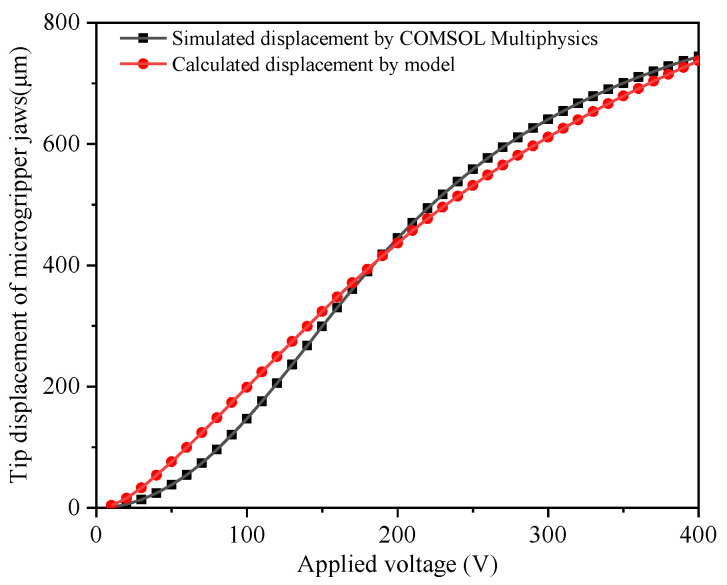
Static performance of the electrostatic repulsive unit in the opto-electrostatic actuator.

Equation (14) represents the static model of the electrostatic repulsive actuator used in the light-operated microgripper. Based on Equation (14), the relationship between the gripping displacement and the applied voltage provided by the PLZT ceramic could be obtained, which shows the actuation characteristic of the proposed opto-electrostaic repulsive actuator, as shown in Figure 14. 

#### 3.2.3. FEA of the Microgripper

The opto-electrostatic repulsive actuator consisted of the PLZT ceramic that provided the photoinduced voltage and the electrostatic repulsive unit that were integral parts of the design of microgripper structures. The microgripper design including the electrostatic repulsive cells was analyzed using finite element analysis in COMSOL Multiphysics software. Due to the geometric symmetry, only half of the microgripper was simulated. The mechanical properties of the materials in models are shown in Table 2. First, the FEM modeling was built through the COMSOL software, which consisted of the clamping arm and the bottom electrodes. Then, the two modules of solid mechanics and electrostatics were selected. The edges of two serpentine springs were fixed constraints, and the voltage was applied by a terminal as designed. The internal air domain was meshed using a free tetrahedral mesh and the other air domain was meshed in a swept way, and the other domain used quadrilateral meshes. After that, the auxiliary sweep with different voltages was used to solve. By applying a voltage as the photoinduced voltage ranging from 0 to 400 V, the tip displacement of the microgripper was simulated. 

**Figure 15:**
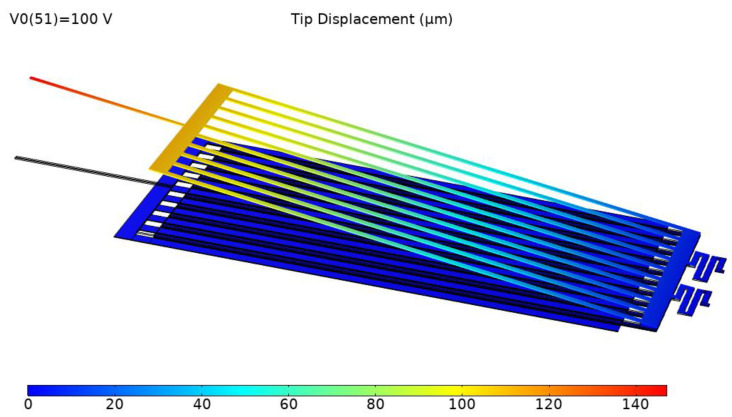
Contour plot of displacement along the *z*-axis.

**Figure 16:**
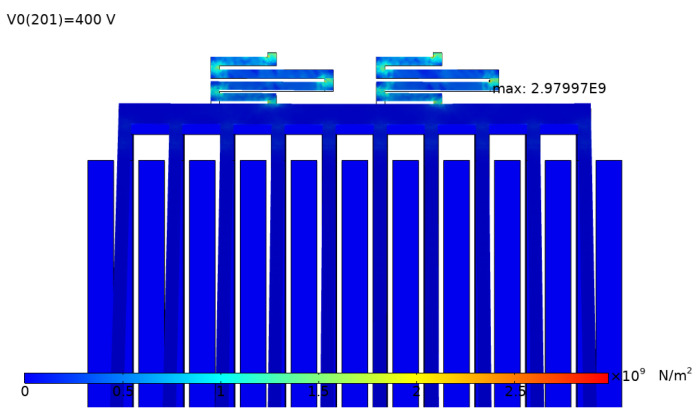
Stress analysis result of the microgripper with the applied voltage of 400 V.

Figure 15 shows the simulation results of the tip displacement of the microgripper jaw with the applied voltage of 100 V. To predict the critical point and the stress distribution of the microgripper, stress analysis was conducted. In the structural design, the clamping arm of the microgripper was made of movable electrodes in electrostatic repulsive cells, which were supported by the supporting spring. The supporting spring bent and generated an elastic restoring force during the operation; therefore, the maximum stress was generated in the supporting spring. Figure 16 shows the stress distribution of the microgripper, from which it is was found that the maximum stress was located on the corner of the supporting spring. The maximum stress with the applied voltage of 400 V was about 2.98 GPa, which is less than the 7 GPa yield strength of single-crystal silicon. 

**Figure 17:**
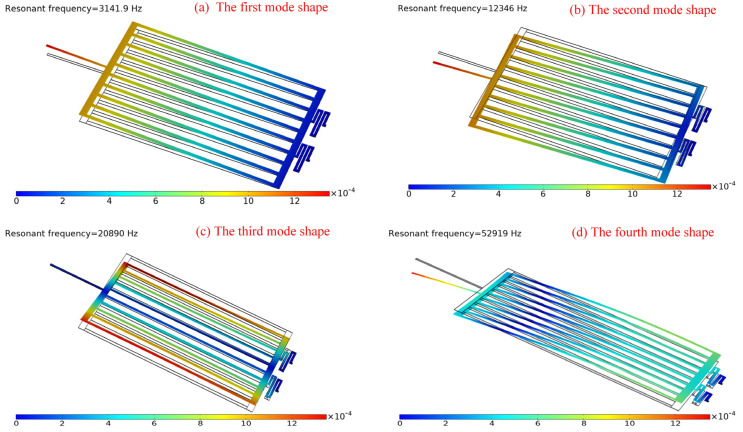
The mode shapes of the microgripper for the first four natural frequencies: (**a**) 3141.9 Hz, (**b**) 12,346 Hz, (**c**) 20,890 Hz and (**d**) 52,919 Hz.

Moreover, to avoid the resonance caused by the coincident vibration frequency and the natural frequency, modal analysis was performed to predict the dynamic performance and the natural frequency of the proposed microgripper. According to the modal analysis, the first four natural frequencies and their mode shapes were obtained, as shown and listed in Figure 17. The results of the model analysis indicated that the resonance phenomenon would not occur because the vibration frequency of an external force was far away from the natural frequency of the microgripper; thus, the microgripper exhibited good dynamic performance.

### 3.3. Static Manipulation Performance of the Light-Operated Microgripper 

According to the above analysis in Section 2.1, the photo-induced voltage of PLZT ceramic could be regarded as the applied voltage of the electrostatic repulsive unit for neglecting the impact of the load. Therefore, the photo-induced voltage could be expressed as shown in our previous works [24]. The relationships between the illumination intensity, time and the tip displacement of the microgripper could be written as follows:

(15) $$V_{s}(1-e^{-\frac{t}{\tau_{1}}})+B_{1}(1-e^{-\frac{t}{\tau_{\theta }}})=\sqrt{\frac{10^{3}D}{NC_{T-D}L\cos\theta \sum_{i=0}^{i=9}A_{i}\int_{L_{0}}^{L_{2}}l{\left(l\sin\theta +d_{2}\right)}^{i}dl}}$$

where $V_{s}$, $\tau_{1}$, $\tau_{\theta }$ and $B_{1}$ are the material performance parameters of PLZT ceramic, whose values are shown in [24]. 

**Figure 18:**
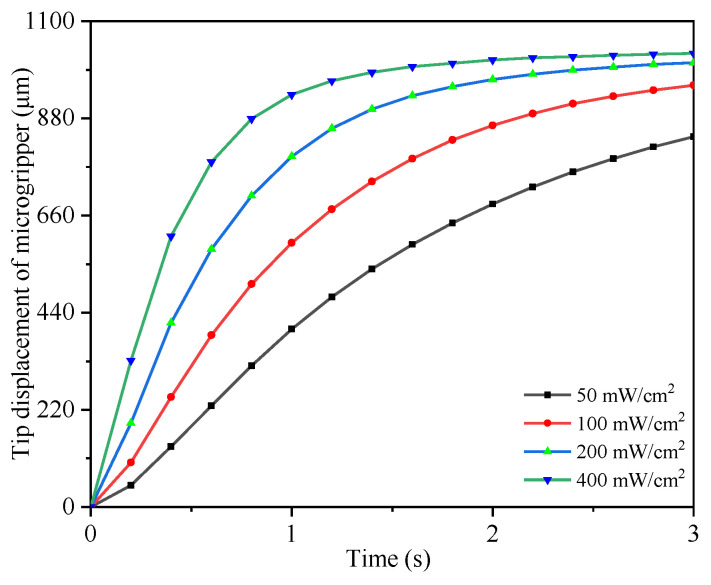
The static performance of the light-operated microgripper under irradiation with different intensities.

Equation (15) shows the static performance of the light-operated microgripper, based on which, the relationships of the tip displacement versus the illumination intensity and time were developed, as shown in Figure 18. Figure 18 illustrates that the response speed and the maximum grasping displacement increased with increasing illumination intensities, from which, it was also revealed that the opto-electrostatic repulsive actuated microgripper could rapidly produce a larger stroke under the irradiation of UV light compared with a traditional electrostatic microgripper. 

## 4. Dynamic Control Experiment of Photovoltage

The driving voltage of the electrostatic repulsive actuator was provided by the PLZT ceramic illuminated by UV light. After confirming the construction and parameters of the 3D model of the microgripper, the static and dynamic performance was determined by the applied voltage. Thus, the dynamic control of the PLZT ceramic was conducted. However, according to our previous study [21], a serious residual photovoltage exists between the two electrodes after the UV light is turned off, which leads to considerable performance implications and a longer response time of the release operation of the microgripper or even causes misoperation. Therefore, not only a stable driving voltage should be acquired but also eliminating the residual photovoltage. 

**Figure 19:**
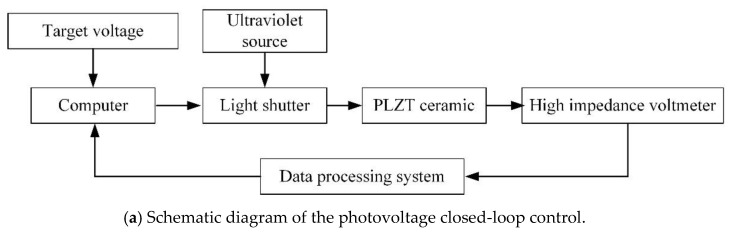

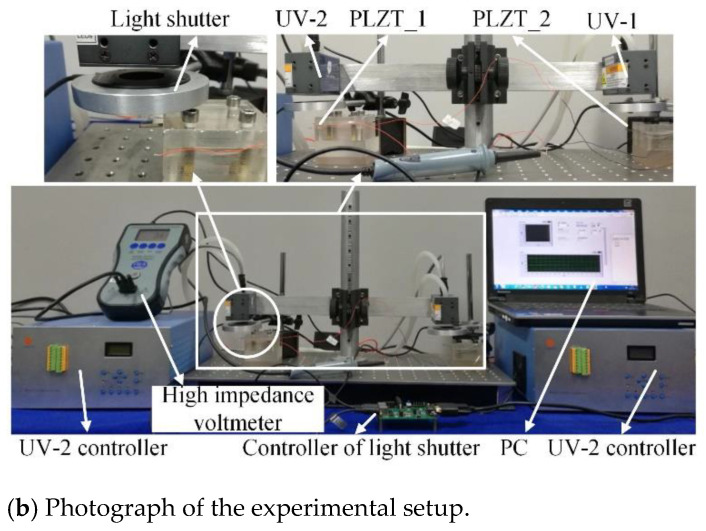
The experimental setup of the photovoltage closed-loop control: (**a**) Schematic diagram of the photovoltage closed-loop control; (**b**) Photograph of the experimental setup.

The photovoltage of a single PLZT ceramic is controlled by controlling the switching of the incident light through the light shutter. The schematic diagram of the photovoltage closed-loop control is shown in Figure 19a. If the measured value was larger than the target value, the light shutter was turned off; otherwise, the light shutter was turned on. Consequently, the photovoltage could be maintained near the target value. The photovoltage values were collected by the high impedance voltmeter, as shown in Figure 19b. 

**Figure 20:**
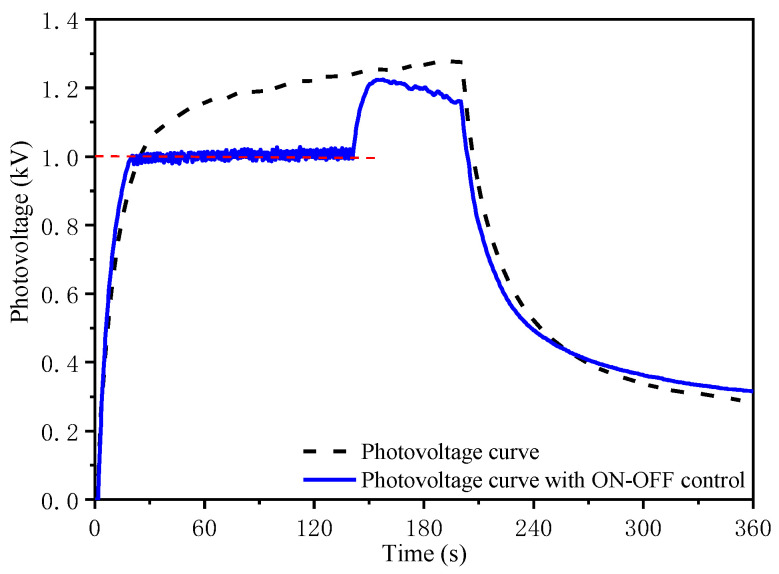
Comparison of photovoltage curves under irradiation of 50 mW/cm^2^ between controlled and uncontrolled photovoltage.

The photovoltage control result of a single PLZT ceramic using an ON–OFF closed-loop control is shown in Figure 20. The black dotted line represents the photovoltage curve under the irradiation of 50 mW/cm^2^, which shows that the photovoltage increased to a steady state and decreased sharply when the UV light was turned off. The blue line shows the photovoltage curve with a target value of 1 kV under the ON–OFF closed-loop control, where the photovoltage stayed around 1 kV and then continued to increase to the maximum value after canceling the ON–OFF control, where the photovoltage also decreases sharply when the UV light was turned off. As can be seen from Figure 20, a simple control method caused the photovoltage of the PLZT ceramic to be stable and suitable as a power supply device, which demonstrated the feasibility of the ON–OFF closed-loop control.

**Figure 21:**
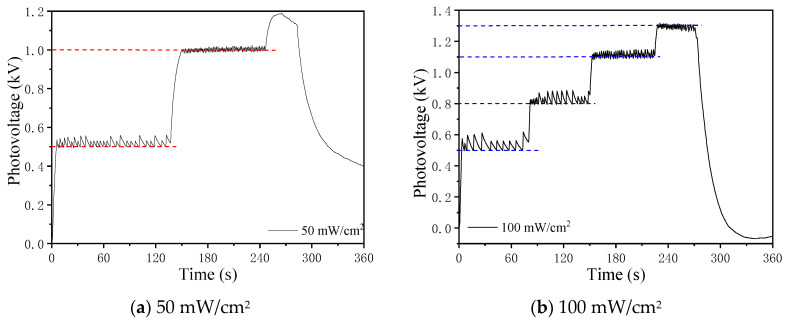
Photovoltage control curves of a single PLZT ceramic with multiple target values under different light intensities: (**a**) 50 mW/cm^2^, (**b**) 100 mW/cm^2^.

Figure 21 shows the control curves of the photovoltage under light intensities of 50 mW/cm^2^ and 100 mW/cm^2^. During the control process, the target values were changed. As shown in Figure 21a, the photovoltage stabilized around the target value of 500 V first, and then remained stable at 1000 V after changing the t74.arget value to 1000 V. Figure 21b gives the control result of photovoltage under the light intensity of 100 mW/cm^2^ with different target values of 500 V, 800 V, 1100 V and 1300 V. The photovoltage under 50 mW/cm^2^ had higher control precision than that under 100 mW/cm^2^, but more than that, the photovoltage with a lower target value also had higher control accuracy than that with a larger target; this phenomenon is thought to be caused by the high response speed of photovoltage, the response speed of the light shutter and the sampling rate of high impedance voltmeter, which did not meet the demand of the response speed of the photovoltage. From Figure 21, the unstable photovoltage phenomena and the residual photovoltage after the ultraviolet source was turned off clearly existed in the test, which had a significant impact on the handling performance of the light-operated microgripper. It is necessary to take measures to flatten the photovoltage fluctuation and eliminate the serious residual photovoltage.

**Figure 22:**
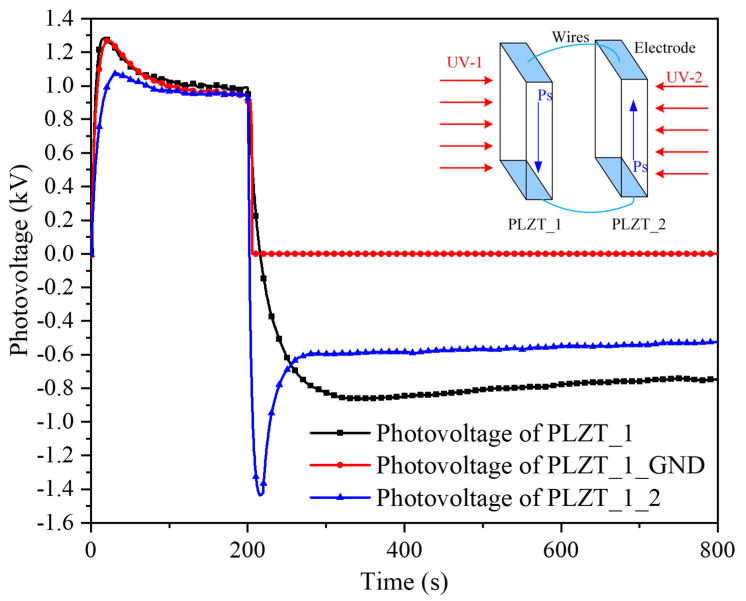
Photovoltage of PLZT_1, PLZT_1_GND and PLZT_1_2 under irradiation of 300 mW/cm^2^.

To eliminate the residual photovoltage, two measures were used, namely, grounding immediately and driving using PLZT_2 after turning off UV-1. Figure 22 illustrates the comparison of the photovoltage of PLZT_1, PLZT_1_GND and PLZT_1_2 in which the PLZT_1 represents the photovoltage of PLZT_1 illuminated by 300 mW/cm^2^ without connecting the PLZT_2, while the PLZT_1_GND expresses the photovoltage of PLZT_1 illuminated by 300 mW/cm^2^, and that was grounded immediately when the UV-1 was switched off. PLZT_1_2 indicates the photovoltage of PLZT_1, which was connected to PLZT_2 with an opposite polarized direction, as shown in the top-right corner of Figure 22, and when UV-1 was turned off, UV-2 was turned on immediately to irradiate PLZT_2, which generated a reversed photovoltage to cancel out the residual direct photovoltage.

Figure 22 shows that a long time was taken to make the residual photovoltage disappear if nothing is done; furthermore, the measures used, namely, grounding and driving by irradiating PLZT_2 with UV-2, were effective at eliminating the residual photovoltage. While driving with PLZT_2 was faster at eliminating the residual photovoltage than grounding, it was difficult to control accurately because of the high response speed of the photovoltage and the PLZT_2 ceramic also generated a reversed residual photovoltage without an effective control strategy. Therefore, as the energy supply device of the microgripper, an ON–OFF closed-loop control method should be adopted to obtain a continuous adjustment photovoltage; in addition, grounding or driving PLZT_2 using UV-2 were used to eliminate the residual photovoltage to achieve a quick release.

## 5. Conclusions

In this study, a novel opto-electrostatic repulsive mechanism and actuator were proposed to provide a solution to realize remote and non-contact light-operated control. The structural design and finite element simulation of the microgripper using an opto-electrostatic repulsive actuator was presented, which has many advantages compared with a microgripper using traditional driving types. The output displacement model of the light-operated microgripper with optimized structure parameters was established and the relationships between the tip displacement and the illumination intensity and time of incident light were obtained. The numeric calculation results were in good agreement with the simulation results, which validated the accuracy of the model of the microgripper and the feasibility and rationality of the proposed structure. The tip displacement of the microgripper was found to approach several hundred micrometers under the irradiation of UV light, where the overall size of the microgripper was 1.3 mm × 0.7 mm × 1.027 mm. The dynamic control experiments of photovoltage showed that the photo-induced voltage of the PLZT ceramic produced a good control effect using an ON–OFF closed-loop control strategy, and some measures should be taken to eliminate the residual photovoltage to achieve a quick release action. Next, the proposed microgripper will be fabricated using PolyMUMPs, and then its static performance will be measured to experimentally validate the accuracy of the output displacement model of the microgripper.

## Figures and Tables

**Table 1:** Structural parameters of the electrostatic repulsive actuator.

Parameter	Symbol	Value	Unit
Lateral distance between adjacent fixed electrodes	*d*_1_	6	μm
Initial vertical distance between moving electrode and central fixed electrode	*d*_2_	1	μm
Width of moving electrode	*W*_m_	15	μm
Width of fixed electrode	*W*_f_	30	μm
Width of central fixed electrode	*W*_fc_	17	μm
Height of moving electrodes	*h*_m_	2	μm
Height of fixed electrodes	*h*_f_	0.5	μm

**Table 2:** Mechanical properties of single-crystal silicon.

Mechanical Properties	Elastic Modulus (GPa)	Poisson’s Ratio	Density (kg/m^3^)	Yield Strength (GPa)
Values	166	0.23	2330	7

## References

[1] Wang D.H., Yang Q., Dong H.M. (2013). A Monolithic Compliant Piezoelectric-Driven Microgripper: Design, Modeling, and Testing. IEEE/ASME Trans. Mechatron..

[2] Das T.K., Shirinzadeh B., Ghafarian M., Al-Jodah A. (2020). Design, analysis, and experimental investigation of a single-stage and low parasitic motion piezoelectric actuated microgripper. Smart Mater. Struct..

[3] Wang F., Liang C., Tian Y., Zhao X., Zhang D. (2015). Design of a Piezoelectric-Actuated Microgripper with a Three-Stage Flexure-Based Amplification. IEEE/ASME Trans. Mechatron..

[4] Chen W., Zhang X., Li H., Wei J., Fatikow S. (2017). Nonlinear analysis and optimal design of a novel piezoelectric-driven compliant microgripper. Mech. Mach. Theory.

[5] Sun X., Chen W., Chen W., Qi S., Li W., Hu C., Tao J. (2019). Design and analysis of a large-range precision micromanipulator. Smart Mater. Struct..

[6] Shen T., Li J., Huang L., Chang J., Xie J. (2019). Dynamic flow characteristics in U-type anti-high overload microfluidic inertial switch. Microfluid. Nanofluid..

[7] Kim D.H., Lee M.G., Kim B., Sun Y. (2005). A superelastic alloy microgripper with embedded electromagnetic actuators and piezoelectric force sensors: A numerical and experimental study. Smart Mater. Struct..

[8] Diller E., Sitti M. (2014). Three-Dimensional Programmable Assembly by Untethered Magnetic Robotic Micro-Grippers. Adv. Funct. Mater..

[9] Chen D.S., Yeh P.F., Chen Y.F., Tsai C.W., Yin C.Y., Lai R.J., Tsai J.C. (2014). An Electrothermal Actuator with Two Degrees of Freedom Serving as the Arm of a MEMS Gripper. IEEE Trans. Ind. Electron..

[10] Solano B., Merrell J., Gallant A., Wood D. (2014). Modelling and experimental verification of heat dissipation mechanisms in an SU-8 electrothermal microgripper. Microelectron. Eng..

[11] Vatan H.M.F., Hamedi M. (2020). Design, analysis and fabrication of a novel hybrid electrothermal microgripper in microassembly cell. Microelectron. Eng..

[12] Chang H., Zhao H., Ye F., Yuan G., Xie J., Kraft M., Yuan W. (2014). A rotary comb-actuated microgripper with a large displacement range. Microsyst. Technol..

[13] Bazaz S.A., Khan F., Shakoor R.I. (2011). Design, simulation and testing of electrostatic SOI MUMPs based microgripper integrated with capacitive contact sensor. Sens. Actuators A Phys..

[14] Jain R.K., Datta S., Majumder S., Dutta A. (2014). Development of multi micro manipulation system using IPMC micro grippers. J. Intell. Robot. Syst..

[15] Cheong H.R., Teo C.Y., Leow P.L., Lai K.C., Chee P.S. (2018). Wireless-powered electroactive soft microgripper. Smart Mater. Struct..

[16] Ford S., Macias G., Lumia R. (2015). Single active finger IPMC microgripper. Smart Mater. Struct..

[17] Shen T., Chang J., Xie J., Huang L. (2020). Analysis of microchannel resistance factor based on automated simulation framework and BP neural network. Soft Comput..

[18] Huang C., Lv J.A., Tian X., Wang Y., Liu J., Yu Y. (2016). A remotely driven and controlled micro-gripper fabricated from light-induced deformation smart material. Smart Mater. Struct..

[19] Yurtsever Z., Küük H., Kucuk H. (2020). Design, Fabrication and Vision Based Operational Analysis of Novel Shape Memory Alloy Micro Grippers. Int. J. Precis. Eng. Manuf..

[20] Garcés-Schröder M., Zimmermann T., Siemers C., Leester-Schädel M., Böl M., Dietzel A. (2019). Shape Memory Alloy Actuators for Silicon Microgrippers. J. Microelectromech. Syst..

[21] Wang X.J., Huang J.H., Wang J. (2015). Experimental research on the response characteristics of PLZT ceramics. Smart Mater. Struct..

[22] Alogla A.F., Amalou F., Balmer C., Scanlan P., Shu W., Reuben R.L. (2015). Micro-Tweezers: Design, Fabrication, Simulation and Testing of a Pneumatically Actuated Micro-Gripper for Micromanipulation and Microtactile Sensing. Sens. Actuators A Phys..

[23] He S., Mrad R.B. (2008). Design, Modeling, and Demonstration of a MEMS Repulsive-Force Out-of-Plane Electrostatic Micro Actuator. J. Microelectromech. Syst..

[24] Huang J.H., Wang X.J., Wang J. (2015). A mathematical model for predicting photo-induced voltage and photostriction of PLZT with coupled multi-physics fields and its application. Smart Mater. Struct..

